# Evaluation of a Trauma-Informed Parenting Program for Resource Parents

**DOI:** 10.3390/ijerph192416981

**Published:** 2022-12-17

**Authors:** Lauren Stenason, Elisa Romano

**Affiliations:** School of Psychology, University of Ottawa, 136 Jean Jacques Lussier, Ottawa, ON K1N 6N5, Canada

**Keywords:** child maltreatment, parenting, child welfare

## Abstract

Child maltreatment impacts many young people involved in the child welfare system, and it is important that the resource parents supporting these youth have knowledge and skills in trauma-informed care. The current study is a preliminary evaluation of the Resource Parent Curriculum (RPC), an in-service, 8-module, group-based parenting program developed by the National Child Traumatic Stress Network. Youth and caregiver outcomes were examined by way of a quasi-experimental design that included 22 resource parents in the experimental group and 21 in the waitlist control group and involved baseline, post-program, and 2-month follow-up assessments. For learning outcomes, RPC resulted in improvements in resource parents’ knowledge and beliefs about trauma-informed parenting. While not statistically significant, potential effects included improvements in resource parents’ tolerance of challenging youth behaviors and parenting self-efficacy. For behavioral outcomes, several non-significant potential effects were noted, including improvements in resource parents’ attachment relationships with their youth and increased social supports. This study was the first to evaluate RPC using a quasi-experimental design within a Canadian context and through a virtual delivery. Findings highlighted several benefits of the program and resource parents’ ongoing training needs.

## 1. Introduction

In Ontario (Canada), the services of child welfare are triggered once a member of the community (a professional or a citizen) makes a report of suspected child maltreatment or risk thereof, which may include physical abuse, neglect, emotional abuse, sexual abuse, concerns regarding caregiver capacity, and/or exposure to intimate partner violence involving a young person under the age of 16 [1,2]. Caregivers may also contact a child welfare agency themselves to seek support for their family. Following a report, an investigation may be conducted with the family, and caseworkers may reach one of three potential conclusions. First, there may be no evidence of maltreatment or future risk of harm, so contact with the family is terminated. Second, there may be no imminent risk of harm to the youth, but the family may be offered supports if they are experiencing difficulties (e.g., counselling, parenting support) [3]. In this case, the youth may also informally reside temporarily with a relative until any safety concerns are resolved. In this arrangement, known as kinship service, the child welfare agency does not have legal custody of the youth. Third, if the youth is at imminent risk of harm, they may be removed from their home and placed in out-of-home care (e.g., foster care, formal kin-based care, or customary care for young people with Indigenous backgrounds). Placement in out-of-home care is not the norm, as data show that the vast majority of youth remain with their families [4].

Young people involved in the child welfare system are at increased risk for physical health problems, poor educational outcomes, mental health difficulties, externalizing behavior, involvement in the justice system, suicidal behavior, and decreased wellbeing overall [5,6,7]. Resource parents (i.e., kinship, foster, and adoptive caregivers, and group home providers) play a crucial role in the lives of young people involved in the child welfare system and require access to effective trauma-informed parenting supports to help these young people heal. They bear much responsibility, as they are tasked with parenting young people who have complex needs, stabilizing their environment, helping ensure they have access to needed services, promoting their physical and mental health and development, navigating the child welfare system, and facilitating connections between the young person and their family of origin [8,9]. To help ensure the recruitment of qualified caregivers, resource parents in Ontario (Canada) must complete several requirements prior to being approved by child protective services. One such requirement in Ontario is pre-service training (i.e., parent training that is completed prior to caring for a young person), which consists of the Parent Resources for Information, Development, and Education (PRIDE) program or, for caregivers of Indigenous young people, the Helping Establish Able Resource-Homes Together (HEART) and Strong Parent Indigenous Relationships Information Training (SPIRIT) [10]. However, there is currently no mandated in-service training (i.e., training that occurs while parenting a young person in care) in Ontario (Canada) for resource parents.

### 1.1. Resource Parent Retention: The Importance of Training

The recruitment and retention of resource parents are key factors in the delivery of quality foster care services [11,12]. Factors related to resource parent retention can be conceptualized at the levels of the child welfare system (e.g., resource parent retention is supported when there is sufficient training and ongoing communication with the child welfare practitioner), resource parent (e.g., social support, confidence, and sense of competence), and youth (e.g., positive youth-caregiver relationship, positive mental health) [11,13,14].

Not only is resource parent retention important, but good-quality resource caregiving is crucial for ensuring positive youth outcomes and resource parent satisfaction [15,16,17]. Young people coming into care experience high levels of developmental trauma, including experiences of child maltreatment. Consequently, it is imperative that resource parents who provide a home for these young people are well supported by their agency and have opportunities to develop competencies in trauma-informed care. When caregivers feel unprepared in caring for youth with high needs, the youth’s mental health difficulties may persist and caregivers may feel less satisfied in their caregiving role, which can contribute to early placement termination and resource parent dropout [18]. In fact, a lack of sufficient training has been cited as one of the main reasons for resource parent dropout, and there is a large unmet need for training and support [19,20]. Resource parent training has been associated with higher parenting skills [21], increased self-efficacy and role satisfaction [22], a willingness to support connections with the youth’s family of origin [17], and fewer reported youth behavior difficulties [23]. Further, effective training of resource parents results in greater retention, placement stability, permanency for youth, and resource parent satisfaction [15,16,17].

### 1.2. Caring for Children Who Have Experienced Trauma: A Workshop for Resource Parents (Resource Parent Curriculum; RPC)

While there are several existing programs, the focus of the current study is on the program, Caring for Children Who Have Experienced Trauma: A Workshop for Resource Parents, otherwise known as the Resource Parent Curriculum (RPC). Kemmis-Riggs and colleagues [24] identified that effective program components of psychological interventions for resource parents include clear program objectives, trauma psychoeducation, attention to specific stages of development, and problem-solving around particular parenting challenges. RPC was selected because it includes all the above program components, is freely available online, and was developed by a reputable source for information on child maltreatment, namely the National Child Traumatic Stress Network (NCTSN).

RPC is an 8-module curriculum that was designed to help therapeutic, foster, adoptive, and kinship caregivers, and group home providers, build their trauma-informed caregiving skills. This program was developed in the United States and is intended for resource parents of youth from birth to age 21. The overall goals of RPC are fourfold: (1) Increase knowledge and beliefs related to trauma-informed parenting; (2) Increase caregivers’ ability to tolerate challenging youth behaviors; (3) Increase parenting self-efficacy; and (4) Increase awareness and the importance of self-care and connections with other resource parents [25]. The eight modules include: Module 1—Welcome and Introductions; Module 2—Trauma 101; Module 3—Understanding Trauma’s Effects; Module 4—Building a Safe Place; Module 5—Dealing with Feelings and Behaviors; Module 6—Connections and Healing, Module 7—Becoming an Advocate, and Module 8—Taking Care of Yourself [26]. Throughout the modules, there are interactive group activities, case examples, and discussions. Previous pre-post evaluations of the RPC program showed that RPC was associated with significant self-reported increases in resource parents’ knowledge and beliefs regarding trauma-informed parenting, parenting self-efficacy, tolerance of challenging behaviors, and recognition of their child’s post-traumatic stress symptoms, as well as decreased parenting stress and negative perceptions of their child [20,27,28,29].

### 1.3. Study Objectives and Hypotheses

The objectives of the current study were to conduct a preliminary evaluation of the Resource Parent Curriculum (RPC). This study consisted of a quasi-experimental design that included within- and between-subject factors. The within-subject factor was time point with three levels (baseline, post-program, and 2-month follow-up), and the between-subject factor was group condition with two levels (waitlist control or experimental). While the larger evaluation included both primary and secondary outcomes, the current study focused on primary outcomes, which consisted of key variables deemed essential to the research question and directly assessed by the RPC program [30].

Outcomes were organized according to Kirkpatrick and Kirkpatrick’s [31] four levels of training evaluation. Level one (reaction) corresponds to the degree to which resource parents found RPC engaging and relevant in their work with young people. These outcomes have been previously reported by Romano and Stenason [32]. Level two (learning) is the degree to which resource parents acquired knowledge, skills, and confidence as a result of their participation in RPC. It was hypothesized that resource parents who participated in RPC would report significantly greater knowledge and beliefs in trauma-informed parenting, greater tolerance of challenging youth behaviors, and improved parenting self-efficacy. It was also expected that these gains would be maintained at the 2-month follow-up. Level three (behavior) is the degree to which resource parents applied what they learned during their participation in RPC. It was hypothesized that resource parents who participated in RPC would report significant improvements in their attachment relationship with their youth, fewer difficult resource parent-youth interactions, and improved social and concrete supports. It was also expected that these gains would be maintained at the 2-month follow-up. Level four (results) is comprised of higher-level, global impacts and was beyond the scope of this preliminary evaluation.

## 2. Methods

### 2.1. Participants

The majority of resource parents (90.5%) identified as female and were 47 years old on average (range 23–70 years). The majority identified as white (90.5%) and married (62.8%). For educational background, most had completed some community college (46.5%) or a bachelor’s or undergraduate degree (23.3%). In terms of work, 39.5% of resource parents identified their main activity as caring for family, and the same percentage identified both caring for family and working for pay/profit (39.5%). While almost 20% of resource parents chose not to disclose their income, just over half reported an income (in Canadian funds) of less than $100,000. The majority of resource parents were foster caregivers (69.7%), followed by adoptive (32.6%), therapeutic (i.e., a form of foster caregiver with specialized training to support youth with complex mental health or medical needs, 27.9%), kinship (9.3%), future adoptive parents (6.9%), and group home providers (4.7%). Note that values exceed 100% because resource parents could select more than one option. The majority of resource parents (79.1%) indicated that they attended the Parent Resources for Information, Development, and Education (PRIDE) training, which is a mandated pre-service training for resource parents in Ontario. Parents identified additional completed trainings, and responses were wide-ranging (e.g., workshops on various mental health challenges). On average, resource parents had been in their role for just under 10 years and had an average of two non-birth children in their care (mean age = 10, range: 5 months to 25 years).

### 2.2. Measures

#### 2.2.1. Resource Parents’ Learning (Level Two)

Knowledge and Beliefs Survey. To assess learning, resource parents completed the Knowledge and Beliefs Survey [29], which is a 33-item questionnaire designed to measure caregiver beliefs and attitudes about caring for youth who have experienced trauma. Items are rated along a 6-point Likert scale from 1 (strongly disagree) to 6 (strongly agree) and measure three subscales: trauma-informed parenting; tolerance of misbehavior (henceforth referred to as tolerance of challenging behaviors); and parenting self-efficacy. For trauma-informed parenting, 24 items assess knowledge about the impact of trauma on youth (e.g., I routinely think about how my child is physically safe in my home, but might not feel safe). This subscale possessed excellent internal consistency in our sample across the three assessments (Cronbach α = 0.90–0.93). The four items on the tolerance of challenging behaviors subscale measure resource parents’ beliefs about their ability to care for youth with behaviors that commonly occur within a trauma context (e.g., I can care for a child who says mean and hurtful things to me). This subscale had good to excellent internal consistency across the three time points (Cronbach α = 0.83–0.92). For parenting self-efficacy, five items assess resource parents’ confidence in their ability to care for a youth with a trauma history (e.g., When things are going badly between my child and me, I keep trying until things begin to change). This subscale demonstrated good to excellent internal consistency across all three time points (Cronbach α = 0.83–0.90).

#### 2.2.2. Resource Parents’ Behavior (Level Three)

Protective Factors Survey. Resource parents completed the Protective Factors Survey—Second Edition (PFS-2) [33], which is a 19-item questionnaire that measures five domains of multi-level family protective factors on a 6-point Likert scale from 0 (not at all like my life) to 5 (just like my life). Four items that measure nurturing/attachment (e.g., I have frequent power struggles with my kids) demonstrated low to adequate internal consistency in our sample across the three time points (Cronbach α = 0.47–0.75). Five items measure social supports (e.g., I have people who believe in me) and had low to adequate internal consistency across the three time points (Cronbach α = 0.66 to 0.79). Four items measure concrete Supports (e.g., I have trouble affording what I need each month) and showed low to adequate internal consistency (Cronbach α = 0.48–0.70). The PFS-2 also includes additional subscales (e.g., family functioning/resilience) which are not reported in the current study, as they were not considered primary evaluation outcomes.

Parenting Stress Index-Short Form, Fourth Edition. Resource parents caring for youth 12 years of age and younger completed the Parenting Stress Index-Short Form, Fourth Edition (PSI-SF) [34]. The PSI-SF is a 36-item measure of parenting stress across three domains. Twelve items measure parent-child dysfunctional interactions (henceforth referred to as resource parent-youth difficult interactions; the quality of the parent-child interactions) and demonstrated good to excellent internal consistency in our sample across the three time points (Cronbach α = 0.85–0.91). The PSI-SF includes additional subscales (i.e., parental distress and difficult child), but they were not considered primary evaluation outcomes.

### 2.3. Procedure

**Figure 1:**
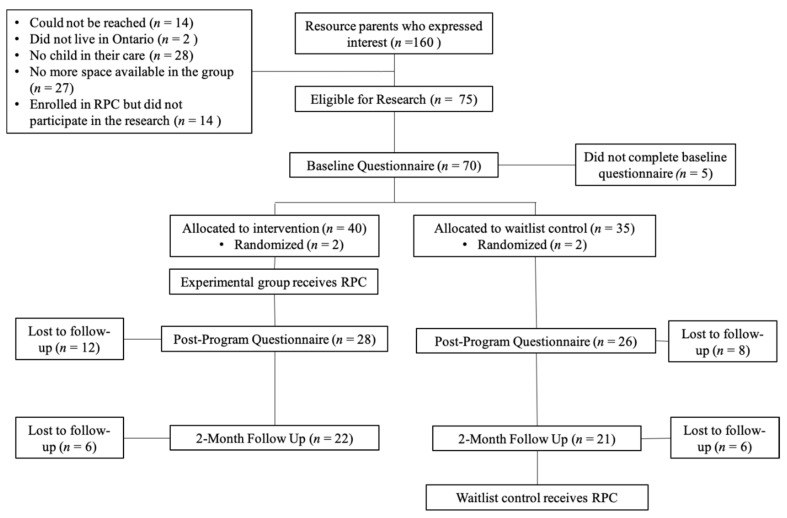
Flow Chart of the Study’s Progress, Including Recruitment, Participation, and Attrition.

Following ethics approval from our university’s research ethics and integrity services, we recruited resource parents through collaboration with two Ontario child welfare agencies and through the community. Resource parents could complete the program without participating in the research. To participate in the RPC program, individuals were required to be a current resource parent in Ontario and have access to a computer or phone with internet. To participate in the research study, they also agreed not to participate in other parenting programs throughout the duration of the research. While group home providers are not traditionally considered resource parents, they nonetheless play a critical role in providing care for youth residing in group home residential placements. Thus, it was deemed important to also offer the intervention to these professionals and include them in the evaluation. To assist them in most accurately completing the measures, group home providers were provided with additional instructions, as some of the measures use language such as “my child”. In particular, they were instructed to consider one particular child with whom they work within the context of their professional role. Figure 1 depicts a consort diagram for participant recruitment and attrition. While the intention was to conduct a randomized controlled trial, only four resource parents agreed to randomization due to scheduling issues. Therefore, the majority of group assignment was non-randomized based on resource parents’ availability.

#### 2.3.1. Data Collection Prior to the Program

Baseline assessment occurred approximately two weeks prior to the beginning of the first module. Resource parents had the option to complete the questionnaire online or by paper. Assessments at the three time points were tracked through a code that resource parents developed. The questionnaire package took approximately 30 min to complete, and resource parents received a $25 gift card for their participation at each data assessment cycle.

#### 2.3.2. Program Delivery

Delivery of the RPC program followed NCTSN implementation guidelines. The program was delivered virtually using a videoconferencing platform due to the COVID-19 pandemic and public health measures. The virtual delivery required only minor modifications to certain activities. The modules were shortened from two hours to 90-minutes in length to reduce barriers to attendance, but all program content was covered. There were six groups in total, including three experimental and three waitlist control groups. The first author was the main facilitator for all six groups. Five of the six groups received eight 90-minute long modules delivered once per week for eight weeks. Due to scheduling constraints, one waitlist control group received 90-minute modules twice per week for four weeks. Five of the six groups were conducted with a co-facilitator with lived experience. There was one group that did not have a co-facilitator due to difficulties finding an available co-facilitator. The facilitator and co-facilitator completed all necessary training available through NCTSN in preparation for facilitating the training.

For the experimental groups, a fidelity monitor (a trained graduate-level research assistant) attended each module to ensure the program was being delivered as intended according to facilitator guidelines. All program components were covered according to the fidelity checklist. Program attendance was also collected for all resource parents. The facilitator contacted all resource parents who missed a module and conducted a phone catch-up call to ensure resource parents were exposed to the material. Approximately 60% of resource parents in the experimental condition attended all eight modules, 27% attended seven, 9% attended six, and 4% attended five modules.

#### 2.3.3. Data Collection following the Program

Within one week after the completion of the training for the experimental group, resource parents in both the experimental and waitlist control groups completed the post-program questionnaire. Two months later, the 2-month follow-up questionnaires were completed. Following this data collection, resource parents in the waitlist control group participated in the RPC program. Two resource parents in the waitlist control group endorsed completing the PRIDE training when asked about participation in other forms of parenting interventions. No resource parents in the experimental condition reported participating in other parenting interventions. While further context or details are not known, depending on the resource parent type, it is possible they had not yet taken PRIDE when they began caring for their youth or were required to take it because it is a ministry requirement for some resource parents in Ontario (Canada).

### 2.4. Statistical Analysis

Questionnaire items were screened prior to statistical analyses for data entry accuracy, missing values, and univariate assumptions. A missing values analysis (MVA) showed that Little’s MCAR test was not significant, and therefore, the data were determined to be missing at random (χ^2^ = 614.96, *p* = 1.00). Missing data ranged from 0 to 4.8% at baseline, post-program, and 2-month follow-up. Missing data were imputed using the expectation-maximization (EM) method. No significant issues with outliers, skewness, or kurtosis were found [35]. The data were also screened for issues with non-linearity, heteroscedasticity, cell numbers, tolerance, and homogeneity of the variance-covariance matrix using scatterplots. The data met all these assumptions according to guidelines by Tabachnick and Fidell [35].

Chi-squared and *t*-test analyses were conducted to determine whether there were any statistically significant differences between the experimental and waitlist control groups on various demographic factors and outcome variables. The only significant difference was that there were significantly more kinship caregivers in the control group (*n* = 4) than in the experimental condition (*n* = 0; χ^2^ = 4.62, *p* = 0.03). Thus, this variable was used as a covariate in all analyses. Overall, these results suggest that the two groups were fairly well matched despite the lack of randomization. For all outcomes reported, the impact of the covariate (i.e., kinship caregivers) was not significant.

A series of univariate analyses were conducted using a repeated-measures General Linear Model. Learning outcomes included knowledge and beliefs regarding trauma-informed parenting, tolerance of challenging behaviors, and parenting self-efficacy. Behavioral outcomes consisted of attachment, social supports, concrete supports, and resource parent-youth difficult interactions. The assumption of sphericity was examined using Mauchley’s Test of Sphericity. If Mauchley’s Test of Sphericity was significant, the assumption of sphericity was violated, and the Huynh-Feld corrected results were interpreted. Significant effects of time were followed up with paired samples *t*-tests, and significant interactions were followed up with independent samples *t*-tests.

Post-hoc exploratory analyses were also conducted for non-significant learning and behavioral outcomes to further explore potential effects given the preliminary nature of this evaluation. Post-hoc exploratory analyses consisted of independent samples and paired samples *t*-tests to examine between- and within-group differences at post-program and the 2-month follow-up. Experiment-wise error was a concern due to the inclusion of a series of analyses. Due to the preliminary nature of this evaluation, results with substantial effect sizes that failed to reach significance were still worth considering as areas to explore in future research [36]. Thus, the results were analyzed both with and without the Bonferroni correction. The non-corrected results were presented, but we have indicated whether the results differed when the Bonferroni correction was applied, using an alpha of 0.007 (0.05/7). Given that the post-hoc analyses were considered exploratory, they were not included in the calculation of the Bonferroni-corrected alpha.

## 3. Results

### 3.1. Resource Parents’ Learning

#### 3.1.1. Trauma-Informed Parenting

**Figure 2:**
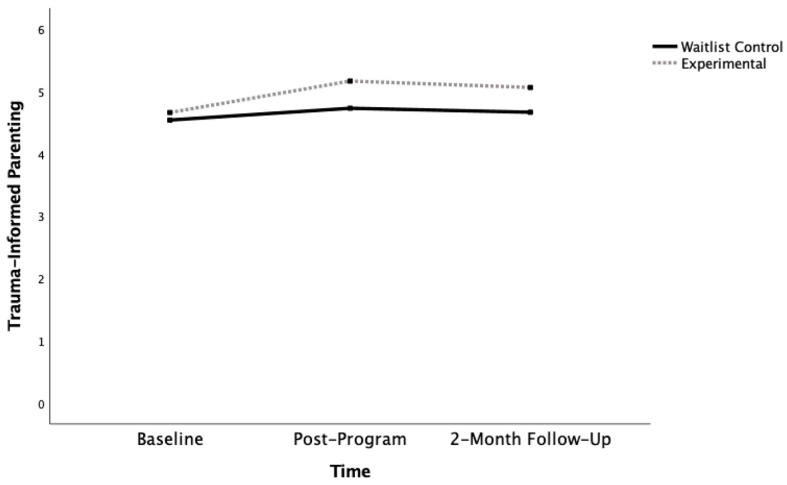
Knowledge and Beliefs in Trauma-Informed Parenting Across Baseline, Post-Program, and 2-Month Follow-Up.

In terms of the General Linear Model, Mauchly’s Test of Sphericity was not significant for trauma-informed parenting (Mauchly’s W = 0.99, *p* = 0.80). The mean baseline scores for trauma-informed parenting were 4.69 for the experimental group and 4.51 for the waitlist control group, which were not significantly different (see Table 1; *t*(41) = −0.81, *p* = 0.21). These scores were generally on the higher end of the 6-point Likert scale. There was a significant impact of time (large effect) and time X group interaction (medium effect; see Table 2 and Figure 2. While the effect of time remained statistically significant, the time X group interaction was no longer statistically significant after applying the Bonferroni correction. For the effect of time, resource parents in the waitlist control reported statistically significant increases in trauma-informed parenting from baseline to post-program (*t*(20) = −2.38, *p* = 0.01), but no significant differences were found between post-program and the 2-month follow-up (*t*(20) = 1.29, *p* = 0.11). Similarly, resource parents in the experimental group also reported significant increases in trauma-informed parenting from baseline to post-program (*t*(21) = −6.37, *p* < 0.001), but no significant differences were found between post-program and the 2-month follow-up (*t*(21) = −0.86, *p* = 0.20). For the interaction, the experimental group reported significantly higher trauma-informed parenting at both post-program (*t*(41) = −3.04, *p* = 0.002) and 2-month follow-up (*t*(41) = −2.37, *p* = 0.01), compared to the waitlist control.

#### 3.1.2. Tolerance of Challenging Behaviors

For the General Linear Model, Mauchly’s Test of Sphericity was not significant (Mauchly’s W = 0.91, *p* = 0.16). The mean baseline scores were 4.30 for the experimental group and 3.99 for the waitlist control group, which were not significantly different (see Table 1; *t*(41) = −1.04, *p* = 0.15). Scores were generally on the higher end of the 6-point Likert scale. There was no significant impact of time (η^2^ = 0.01, small effect) or time × group interaction (η^2^ = 0.05, small effect; see Table 2). For the post-hoc exploratory analyses, resource parents in the waitlist control group did not report changes to their tolerance of challenging behaviors from baseline to post-program (*t*(20) = 0.22, *p* = 0.42) or from post-program to 2-month follow-up (*t*(20) = −0.88, *p* = 0.19). Conversely, those in the experimental group reported significant increases in their tolerance of challenging behaviors from baseline to post-program (*t*(21) = −2.48, *p* = 0.01) but not from post-program to 2-month follow-up (*t*(21) = 0.69, *p* = 0.25). For potential between-group differences, despite no statistically significant baseline differences, the experimental group reported a significantly higher tolerance of challenging behaviors at post-program compared to the waitlist control (*t*(41) = −2.07, *p* = 0.02). However, the scores for the two groups did not differ significantly by the 2-month follow-up (*t*(41) = −1.60, *p* = 0.06).

#### 3.1.3. Parenting Self-Efficacy

For the General Linear Model, Mauchly’s Test of Sphericity was significant (Mauchly’s W = 0.82, *p* = 0.02), and the Huynh-Feldt corrected results were used. The mean baseline scores for parenting self-efficacy were 4.86 for the experimental group and 4.66 for the waitlist control group, which were not significantly different (see Table 1; *t*(41) = −0.81, *p* = 0.21). Scores were on the higher end of the 6-point Likert scale. There was no significant impact of time (η^2^ = 0.04, small effect) or time × group interaction (η^2^ = 0.04, small effect; see Table 2).

In terms of the post-hoc exploratory analyses, resource parents in the waitlist control group reported no significant changes in their parenting self-efficacy from baseline to post-program (*t*(20) = −0.28, *p* = 0.39) or from post-program to 2-month follow-up (*t*(20) = 0.00, *p* = 0.50). Conversely, resource parents in the experimental group reported significant increases in their parenting self-efficacy from baseline to post-program (*t*(21) = −2.88, *p* = 0.01) but not from post-program to 2-month follow-up (*t*(21) = −0.57, *p* = 0.29). For potential between-group differences, despite no statistically significant baseline differences between the experimental and waitlist control groups, resource parents in the experimental group reported significantly higher parenting self-efficacy at post-program (*t*(41) = −2.39, *p* = 0.01) and at 2-month follow-up (*t*(41) = −3.05, *p* = 0.00), compared to those in the waitlist control.

### 3.2. Resource Parents’ Behavior

#### 3.2.1. Attachment

For the General Linear Model, Mauchly’s Test of Sphericity was not significant (Mauchly’s W = 0.96, *p* = 0.41). The mean baseline scores were 2.71 for the experimental group and 2.49 for the waitlist control group, which were not significantly different (see Table 1; *t*(41) = −1.05, *p* = 0.15). Scores fell within the mid-range of the 5-point Likert scale. Table 2 shows that there was no significant impact of time (η^2^ = 0.00, small effect) or time × group interaction (η^2^ = 0.04, small effect). For the post-hoc exploratory analyses, resource parents in the waitlist control group reported no changes in their attachment relationship with the youth from baseline to post-program (*t*(20) = −1.05, *p* = 0.15) or from post-program to 2-month follow-up (*t*(20) = 1.59, *p* = 0.06). Although resource parents in the experimental group reported no significant changes in their attachment relationship with the youth from baseline to post-program (*t*(21) = 0.54, *p* = 0.30), they did report significant improvements from post-program to the 2-month follow-up (*t*(21) = −1.81, *p* = 0.04). For potential group differences, the two groups did not differ significantly at post-program (*t*(41) = −0.28, *p* = 0.39). However, those in the experimental group reported significantly higher attachment than those in the control group at the 2-month follow-up (*t*(41) = −1.77, *p* = 0.04).

#### 3.2.2. Social Supports

For the General Linear Model, Mauchly’s Test of Sphericity was not significant (Mauchly’s W = 0.91, *p* = 0.17). The mean baseline scores were 3.04 for the experimental group and 3.06 for the waitlist control group, which were not significantly different (see Table 1; *t*(41) = 0.10, *p* = 0.46). Scores generally fell within the mid to high end of the 5-point Likert scale. There was no significant effect of time (η^2^ = 0.03, small effect) or time × group interaction (η^2^ = 0.03, small effect; see Table 2). For the post-hoc exploratory analyses, resource parents in the waitlist control group reported no significant changes in their social supports from baseline to post-program (*t*(20) = −0.62, *p* = 0.27) or from post-program to 2-month follow-up (*t*(20) = 1.30, *p* = 0.10). Resource parents in the experimental condition reported significant changes in social supports from baseline to post-program (*t*(21) = -2.33, *p* = 0.02), but no significant changes were found from post-program to 2-month follow-up (*t*(21) = 0.33, *p* = 0.37). For potential between group differences, although the two groups did not differ significantly at post-program (*t*(41) = −0.29, *p* = 0.29), those in the experimental group reported significantly higher social supports at the 2-month follow-up (*t*(41) = −1.82, *p* = 0.04).

#### 3.2.3. Concrete Supports

For the General Linear Model, Mauchly’s Test of Sphericity was significant (Mauchly’s W = 0.76, *p* = 0.01), so the Huynh-Feldt corrected results were used. The mean baseline scores were 3.80 for both the experimental group and waitlist control group and were not significantly different (see Table 1; *t*(41) = −0.02, *p* = 0.49). Scores fell within the mid to high end of the 5-point Likert scale. There was a significant effect of time (η^2^ = 0.08, medium effect), but no significant time × group interaction (η^2^ = 0.05, small effect; see Table 2). In terms of the significant effect of time, resource parents in the waitlist control group reported a significant decrease in their concrete supports from baseline to post-program (*t*(20) = 2.12, *p* = 0.02), but no significant differences were found from post-program to 2-month follow-up (*t*(20) = −1.60, *p* = 0.06). On the other hand, resource parents in the experimental group did not report any significant changes in their concrete supports from baseline to post-program (*t*(21) = 0.39, *p* = 0.35) or from post-program to 2-month follow-up (*t*(21) = −1.05, *p* = 0.15). For the post-hoc exploratory analysis, potential between group differences were examined. The two groups did not differ significantly at post-program (*t*(41) = −1.21, *p* = 0.12) or at 2-month follow-up (*t*(41) = −1.11, *p* = 0.14).

#### 3.2.4. Resource Parent-Youth Difficult Interactions

For the General Linear Model, Mauchly’s Test of Sphericity was not significant (Mauchly’s W = 0.93, *p* = 0.54). The mean baseline scores were 28.60 for the experimental group and 31.80 for the waitlist control group, which were not significantly different (see Table 1; *t*(18) = 0.77, *p* = 0.23). Scores fell within the mid-range of the overall scale. There was a significant impact of time (η^2^ = 0.20, large effect) but no significant time X group interaction (η^2^ = 0.04, small effect; Table 2). For the significant impact of time, resource parents in the waitlist control did not report significant decreases in their difficult interactions with youth from baseline to post-program (*t*(9) = −0.07, *p* = 0.47) but did report a decrease from post-program to 2-month follow-up (*t*(9) = 2.67 *p* = 0.01). Resource parents in the experimental group did not report a significant change in their difficult interactions with youth from baseline to post-program (*t*(9) = 1.23, *p* = 0.13) or from post-program to 2-month follow-up (*t*(9) = 1.42, *p* = 0.09). A post-hoc analysis for potential differences between groups was conducted. The two groups did not differ significantly at post-program (*t*(18) = 1.39, *p* = 0.09) or at the 2-month follow-up (*t*(18) = 1.48, *p* = 0.08).

## 4. Discussion

### 4.1. Preliminary Effects of RPC on Resource Parents’ Learning

The hypotheses about group changes from baseline to post-program, as well as from post-program to the 2-month follow-up, were partially supported. The results showed that, compared to the waitlist control, resource parents who participated in RPC developed greater knowledge and beliefs about trauma-informed parenting, which was a medium effect. These knowledge gains were maintained two months after the program’s completion. Resource parents who participated in RPC were no more likely than those in the waitlist control group to experience increases in their tolerance of challenging behaviors or parenting self-efficacy. However, post-hoc exploratory analyses for post-program results showed potential effects on increased tolerance of challenging behaviors and parenting self-efficacy for resource parents who participated in RPC compared to the waitlist control. However, these gains were only maintained at the 2-month follow-up for parenting self-efficacy.

These findings are somewhat in line with previous RPC evaluations using the same measure. Namely, Sullivan and colleagues [29] found that resource parents showed significant increases in their knowledge of trauma-informed parenting and parenting self-efficacy from baseline to post-program. Although non-kinship caregivers reported increases in their tolerance of challenging youth behaviors, kinship caregivers did not report such changes. A more recent pre-post evaluation also found that resource parents who participated in RPC showed improvements in their knowledge of trauma-informed parenting, parenting self-efficacy, and tolerance of challenging youth behaviors [20]. It appears that the most consistent findings pertain to trauma-informed parenting, with greater inconsistency for parenting self-efficacy and tolerance of challenging behaviors. Given that RPC’s learning objectives focus heavily on the acquisition of knowledge related to trauma-informed parenting, findings from the current study provide strong support for this particular domain. It should be noted that previous RPC evaluations did not include a comparison group. Solomon and colleagues [23] noted that relatively few studies have a comparison group, and as a result, there are limitations in interpreting the program’s effectiveness.

One possible explanation for the low efficacy was the low observed power, which may have decreased the chances of statistically significant findings. There was also a sizeable proportion of resource parents who self-identified as therapeutic/treatment foster caregivers. While there were no significant differences in the number of therapeutic/treatment foster caregivers between the experimental and waitlist control groups, the higher proportion of therapeutic/treatment foster caregivers may have contributed to a potential “ceiling effect” given that this group may already have a high degree of knowledge regarding trauma-informed parenting. Indeed, resource parents in the current sample had been in their role for 10 years on average, and the low efficacy of the intervention may be explained by this ceiling effect given scores fell on the higher end of the scales across outcomes. Another possible consideration involves the impact of COVID-19, as the program was delivered during a time of high global stress that directly impacted families. Although resource parents’ responses on outcome variables were generally on the higher end of the scales, resource parents were known to face added stressors that included communication difficulties with child welfare staff, reduced mental health and training supports, and disruptions in visitation or access visits with biological families [37,38,39]. Given these stressors, if resource parents were struggling to care for their youth with high mental health needs during a time when they were receiving limited supports and services, they may have felt less competent and confident in their caregiving role, and less equipped to persist through difficulties, thereby impacting their parenting self-efficacy and tolerance of challenging behaviors.

### 4.2. Preliminary Effects of RPC on Resource Parents’ Behavior

The hypotheses related to attachment, social supports, concrete supports, and resource parent-youth difficult interactions were not supported. Results showed that RPC did not significantly improve the resource parent-reported attachment relationship with their youth, social supports, concrete supports, or difficult resource parent-youth interactions. Post-hoc exploratory analyses indicated potential effects in some of these areas. In particular, while scores for the two groups were equivalent at post-program, resource parents in the experimental group reported a significantly higher quality attachment relationship with their youth and higher social supports at the 2-month follow-up compared to the waitlist control. While resource parents in the waitlist control group reported decreases in their concrete supports from baseline to post-program, scores for the two groups did not differ significantly at post-program or the 2-month follow-up. Finally, resource parents in the waitlist control group reported significant decreases in their difficult interactions with their youth from post-program to the 2-month follow-up, while resource parents in the experimental group did not report such changes. However, the two groups did not differ significantly at the post-program or 2-month follow-up.

Previous research related to the effectiveness of resource parent interventions has been inconsistent. While RPC has not been previously evaluated using the behavioral outcomes included in the current study, results can be compared to research investigating similar outcomes for other resource parent interventions. The results of the current study were fairly inconsistent with a series of eight meta-analyses conducted by Schoemaker et al. [40] examining the effectiveness of intervention programs for kinship, foster, and adoptive caregivers across several outcomes that included sensitive parenting, dysfunctional discipline, parenting stress, and attachment security. The results indicated positive and statistically significant impacts for sensitive parenting, dysfunctional discipline, and parenting stress but not for attachment security. Improvements in sensitive parenting were larger if the intervention was delivered to groups versus individuals, with even greater improvements when the intervention was delivered in groups with additional individual sessions [40]. It is possible that resource parents in the current study may have required additional individualized or intensive resources by way of individual sessions to continue supporting their parenting work, and that this added component may have resulted in statistically significant outcomes at the behavioral level (e.g., improved resource parent-youth interactions). Furthermore, resource parents were facing reduced support throughout the duration of the study due to the COVID-19 pandemic. Thus, it is possible that services around respite, mental health, and school-based supports were eliminated or significantly reduced throughout the time that the program was delivered. For many families, it is possible that RPC was the only support they were receiving, and the program may not have adequately addressed the full range of parenting and mental health needs necessary for supporting youth and caregivers.

The current study was generally consistent with previous literature related to attachment, as a meta-analysis of six studies found that, with the exception of one study, resource parent interventions overall were not effective at improving attachment security [40]. A potential effect in the current study was found through post-hoc exploratory analyses, as resource parents in the experimental group reported a significantly higher quality attachment relationship with their youth at the 2-month follow-up compared to the waitlist control. In general, measures related to attachment are not commonly included in evaluations of resource parent programs, which is problematic given the high prevalence of attachment difficulties for youth in care [41]. Perhaps resource parent interventions targeting the attachment relationship may be more effective for particular age groups (e.g., early childhood) due to sensitive periods of development. Attachment security for older youth may be more challenging to target through a group-based caregiver program if youth have experienced high levels of placement instability and/or more chronic maltreatment. It is possible that more comprehensive, extensive supports of a longer duration may be needed to improve the quality of youth attachment relationships in such circumstances. Healing from child maltreatment is a long-term process, and the therapeutic benefits to the youth are unlikely to be seen without a long period of stability and predictability [42]. Thus, it is understandable that RPC in its current 8-module format would not fully address the complexities of youth attachment challenges. Indeed, Schoemaker et al. [40] noted that indirect youth-level outcomes may be delayed and suggested that long-term follow-up (i.e., longer than 6 months) may be necessary to examine these effects. In the current study, resource parents in the experimental group only began reporting improvements in the attachment relationship with their youth at the 2-month follow-up, so it is possible that these changes may continue to develop over time.

### 4.3. Limitations and Future Research Directions

There are a number of limitations to the current study. In terms of design, resource parents self-selected to participate in RPC and were not randomly selected from the population of all Ontario resource parents. The majority of resource parents were not randomized to experimental or waitlist control groups. Future evaluations should strive for random assignment to groups, which would result in a greater likelihood of true group equivalence and superior generalizability. Additionally, given the novel virtual delivery of RPC, it is not known how these adaptations impacted the program’s effectiveness, as all previous evaluations of RPC were based on in-person delivery.

Due to the preliminary nature of the evaluation and the challenges of intervention research (especially during a pandemic), the sample size was small, and there were multiple comparisons, so the analyses were underpowered. It would be important for future evaluations to have a larger sample to follow-up on some of the potential effects that were found through post-hoc exploratory analyses. Important areas to further examine may include whether results differed by resource parent type, the dosage and timing of content, setting or format, and the specific intervention ingredients that are most impactful. These dismantling studies are necessary to determine which components of the program are most effective [40].

Another limitation related to data collection was that responses were based solely on resource parent self-reports. Gathering perspectives from multiple informants (e.g., the youth themselves, mental health records, or school records) would have increased the validity of the findings. There were also several measures that had poor internal consistency, which can be impacted by the number of items in a questionnaire, the sample size, and the level of homogeneity in the sample (e.g., if resource parents’ responses were homogeneous and had low standard deviations) [43]. In addition, some of the measures were designed for parents and not a resource parent population (e.g., PSI, PFS). While group home providers were instructed to respond based on a youth with whom they currently work, it is possible that they may have approached the measure differently than the original population for which the measure was intended. Finally, it was not possible to examine more distal outcomes including placement disruption. These more distal outcomes, would be critical to examine, given previous research that has found links between resource parent training and placement stability, resource parent retention, and satisfaction [44,45].

In terms of the next steps, RPC is over ten years old, so updates are underway to reflect current knowledge in the field. Currently, RPC does not explicitly address how diversity-related factors such as race, gender identity, sexual orientation, religion, and socioeconomic status impact family systems, caregivers, and youth in care. Given the overrepresentation of Black and Indigenous youth in the Ontario child welfare system [46], updates to the RPC program must address these factors so that resource parents can better understand the impact of systemic oppression and the lived experiences of individuals within the child welfare system. It is critical that future research be conducted evaluating the program as possible changes are made to program content. Future research should also include a more diverse sample of resource parents.

## 5. Conclusions

Young people in care cope with high levels of adversity and mental health challenges due to the impact of child maltreatment. The resource parents caring for these youth receive limited support and resources, yet are tasked with many important responsibilities in caring for youth with complex needs. The current study is an important first step toward evaluating RPC using more rigorous methods using a quasi-experimental design within a Canadian context and using a virtual format.

While it is important that resource parents be satisfied with the RPC program [32], they must also have the opportunity to develop greater competencies, including knowledge and skill acquisition [47]. Preliminary outcome findings suggest that RPC has the potential to improve resource parent knowledge and beliefs regarding trauma-informed parenting and highlight potential areas to further explore as they relate to attachment and social supports. Resource parents and youth in care require a comprehensive approach to service delivery, as RPC alone was not enough to address outcomes at the behavioral level (e.g., improved interactions between the resource parent and youth) and did not fully address their complex needs. A multi-pronged approach embedded in a trauma-informed system where all individuals have their needs met (i.e., youth, resource parents, biological families, child welfare practitioners) is critical when addressing complex issues related to supporting youth and resource parents.

While in-service supports provide resource parents with the necessary foundational knowledge prior to caring for the young person, they must also receive in-service supports that are trauma-informed. Despite this need for in-service supports, there were barriers to partnering with child welfare agencies during recruitment. In fact, the majority of resource parents who expressed interest in the program sought this opportunity outside of their child welfare agency. This speaks to the ongoing training needs of resource parents and suggests there may not be sufficient parenting supports offered within their child welfare agency. Novel programs can be difficult to introduce and implement in organizations due to complex and layered contexts, limited resources, and high staff turnover [48,49,50]. Furthermore, the COVID-19 pandemic had a significant impact on the supports and services offered by child welfare organizations, so it is understandable that as recruitment progressed throughout the duration of the pandemic, resource parents sought additional supports and services in the community. The pandemic also drove novel adaptations to the program’s delivery, which may serve to increase access and reduce barriers to attendance. Overall, healing for young people with histories of child maltreatment can only occur within the context of consistent, stable, and nurturing relationships, so it is critical that resource parents be supported in their caregiving role.

## Figures and Tables

**Table 1:** Descriptive Statistics for Outcome Variables.

	Theoretical Range	Baseline		Post-Program		2-Month Follow-up	
		Experimental	Waitlist Control	Experimental	Waitlist Control	Experimental	Waitlist Control
		(M, SD), *n*	(M, SD), *n*	(M, SD), *n*	(M, SD), *n*	(M, SD), *n*	(M, SD), *n*
Resource Parents’ Learning							
Trauma-Informed Parenting	1–6	4.69 (0.58), 22	4.51 (0.79), 21	5.17 (0.40), 22	4.73 (0.54), 21	5.09 (0.62), 22	4.64 (0.63), 21
Tolerance of Challenging Behaviors	1–6	4.30 (0.92), 22	3.99 (0.98), 21	4.63 (0.75), 22	3.94 (1.33), 21	4.56 (0.79), 22	4.13 (0.94), 21
Parenting Self-Efficacy	1–6	4.86 (0.61), 22	4.66 (0.97), 21	5.17 (0.51), 22	4.71 (0.73), 21	5.24 (0.52), 22	4.71 (0.60), 21
Resource Parents’ Behavior							
Attachment	1–5	2.71 (0.68), 22	2.49 (0.68), 21	2.64 (0.58), 22	2.60 (0.57), 21	2.80 (0.57), 22	2.43 (0.76), 21
Social Supports	1–5	3.04 (0.68), 22	3.06 (0.62), 21	3.31 (0.59), 22	3.18 (0.87), 21	3.27 (0.58), 22	2.94 (0.61), 21
Concrete Supports	1–5	3.80 (0.37), 22	3.80 (0.28), 21	3.77 (0.39), 22	3.58 (0.62), 21	3.83 (0.29), 22	3.70 (0.44), 21
Resource Parent-Youth Difficult Interactions Percentile Rank ^a^	12–60	28.60 (8.37), 10 76th	31.80 (10.22), 10 82nd	26.10 (8.11), 10 66th	31.90 (10.42), 10 82nd	23.30 (9.43), 10 54th	29.40 (9.06), 10 62nd

^a^ 16th–84th percentiles = Normal range; 85th–89th percentiles = High range; 90th percentile or higher = Clinically significant range. Normative data were collected from a sample of 534 mothers and 522 fathers stratified to match U.S 2007 Census.

**Table 2:** Results for the RPC’s Impact on Preliminary Outcomes of Resource Parents’ Learning and Behavior.

	*n*						
	Waitlist Control	Experimental	F	*p*	η^2^	95% CI for η^2^	Observed Power
Resource Parents’ Learning							
Trauma-Informed Parenting	21	22					
Time			15.09	<0.001	0.27	[0.06, 0.45]	0.99
Time × Group interaction			3.77	0.03	0.09	[0.00, 0.26]	0.67
Tolerance of Challenging Behaviors	21	22					
Time			0.51	0.60	0.01	[0.00, 0.14]	0.13
Time × Group interaction			2.30	0.11	0.05	[0.00, 0.22]	0.45
Parenting Self-Efficacy	21	22					
Time			1.82	0.17	0.04	[0.00, 0.20]	0.35
Time × Group interaction			1.71	0.19	0.04	[0.00, 0.19]	0.34
Resource Parents’ Behavior							
Attachment	21	22					
Time			0.06	0.94	0.00	[0.00, 0.06]	0.06
Time × Group interaction			1.76	0.18	0.04	[0.00, 0.19]	0.36
Social supports	21	22					
Time			1.20	0.31	0.03	[0.00, 0.18]	0.26
Time × Group interaction			1.13	0.33	0.03	[0.00, 0.17]	0.24
Concrete supports	21	22					
Time			3.67	0.04	0.08	[0.00, 0.26]	0.62
Time × Group interaction			2.10	0.14	0.05	[0.00, 0.21]	0.39
Resource parent-youth difficult interactions	10	10					
Time			4.33	0.02	0.20	[0.00, 0.27]	0.71
Time × Group interaction			0.79	0.46	0.04	[0.00, 0.16]	0.17

Note: η^2^, values between 0.01 and 0.05 represent a small effect, values between 0.06 and 0.13 represent a medium effect, and values greater than 0.14 represent a large effect.

## Data Availability

Data sharing is not applicable to this article because participants did not consent to their data being shared with individuals outside of the research team.
